# Supplementary material for: Metabolic Risk Factors for Hepatocellular Carcinoma in Patients with Nonalcoholic Fatty Liver Disease: A Prospective Study
Source: Cancers (Basel). 2022 Dec 17;14(24):6234. doi: 10.3390/cancers14246234 (PMC9777437; doi:10.3390/cancers14246234)
Supplement: Supplementary file 1 [file cancers-14-06234-s001.zip › cancers-2019732-supplementary.pdf]

Supplementary Materials: Metabolic Risk Factors for Hepatocellular Carcinoma in Patients with Nonalcoholic Fatty Liver Disease: A Prospective Study

Samuel O. Antwi, Emily C. Craver, Yvonne A. Nartey, Kurt Sartorius and Tushar Patel

Table S1. International Classification of Diseases, ninth and tenth revisions, Clinical Modification (ICD-9-CM and ICD-10-CM) codes used in the study.

Condition	ICD-9 codes	ICD-10 codes
Included Conditions		
Non-alcoholic fatty liver Disease (NAFLD)	571.8	K76.0
Any cirrhosis	571.5, 456.1, 456.21,456.0, 456.20, 789.5, 572.2	K74.6, I85.9, I98.2, I86.4, I85.0, I98.3, R18
Excluded Conditions		
Alcoholic liver disease (ALD)	571.0-571.3	K70
Alcoholic use disorder /alcohol abuse	291.0–291.3, 291.5, 291.8, 291.9, 303.x, 305.0	F10
Somatic consequences of alcohol	291, 357.5, 425.5, 535.3, 980.1, 980.9	E24.4, G62.1, I42.6, K29.2, G31.2, G72.1, K85.2, K86.0, T51.0, T51.9, Y57.3, X65, Z50.2, Z71.4, Z72.1
Viral hepatitis B and C	070.xx, V0261, V02.62	B16, B17, B18, B19
Autoimmune liver disease (autoimmune hepatitis, primary biliary cirrhosis, primary sclerosing cholangitis)	571.6, 576.1	K83.0A, K83.0F, K74.3, K75.4
Hemochromatosis	275.0	E83.1
Wilson's disease	275.1	E83.0B
Alpha-1-antitrypsin deficiency	277.6	E88.0A, E88.0B
Budd-Chiari syndrome	453.0	I82.0, K76.5
Chronic hepatitis, unspecified	571.4	K73.9, K73.2
Secondary or unspecified biliary cirrhosis	571.6	K74.4, K74.5
Drug use disorders (except nicotine and caffeine)	305.1-9	F11-F14, F16, F18, F19
Biliary cirrhosis	571.6	K74.3
Variables		
Diabetes mellitus	250.xx	E10.x–E14.x
Obesity	278.0, 278.1,278.01, 278.00	E66.x
Dyslipidemia	272.0, 272.1, 272.2, 272.4, 272.5, 272.7, 272.9	E78.0, E78.2, E78.4, E78.41, E78.49, 78.5.
Hypertension	401.x, 402.x, 403.x, 404.x, 405.x	I10.x, I11.x–I13.x, I15.x
Hypothyroidism	240.9, 243.x, 244.x, 246.1, 246.8	E00.x–E03.x, E89.0
Smoking history/tobacco use	V15.82, 305.1, 989.84	Z72.0, Z87.891

ICD-codes for selection of the inclusion and exclusion criteria, and well as the metabolic conditions were obtained from Hagström H, Adams LA, Allen AM, et al. Administrative Coding in Electronic Health Care Record-Based Research of NAFLD: An Expert Panel Consensus Statement. Hepatology 2021;74(1):474-482. Other ICD codes for SEER covariates data, e.g., smoking and hypertension, were based on codes from Welzel TM, Graubard BI, Zeuzem S, El-Serag HB, Davila JA, McGlynn KA. Metabolic syndrome increases the risk of primary liver cancer in the United States: a study in the SEER-Medicare database. Hepatology. Aug 2011;54(2):463-71; and Kanwal F, Kramer JR, Mapakshi S, et al. Risk of Hepatocellular Cancer in Patients with Non-Alcoholic Fatty Liver Disease. Gastroenterology. Dec 2018;155(6):1828-1837.e2.
